# A curated dataset of modern and ancient high-coverage shotgun human genomes

**DOI:** 10.1038/s41597-021-00980-1

**Published:** 2021-08-04

**Authors:** Pierpaolo Maisano Delser, Eppie R. Jones, Anahit Hovhannisyan, Lara Cassidy, Ron Pinhasi, Andrea Manica

**Affiliations:** 1 Department of Zoology, University of Cambridge, Cambridge, CB2 3EJ UK; 2 Smurfit Institute of Genetics, Trinity College Dublin, Dublin, 2 Ireland; 3 Genomics Medicine Ireland, Dublin, Ireland; 4 Institute of Molecular Biology, National Academy of Sciences, 7 Hasratyan Street, 0014 Yerevan, Armenia; 5 Department of Evolutionary Anthropology, University of Vienna, 1090 Vienna, Austria

**Keywords:** Population genetics, Genetic variation, Genetic variation

## Abstract

Over the last few years, genome-wide data for a large number of ancient human samples have been collected. Whilst datasets of captured SNPs have been collated, high coverage shotgun genomes (which are relatively few but allow certain types of analyses not possible with ascertained captured SNPs) have to be reprocessed by individual groups from raw reads. This task is computationally intensive. Here, we release a dataset including 35 whole-genome sequenced samples, previously published and distributed worldwide, together with the genetic pipeline used to process them. The dataset contains 72,041,355 sites called across 19 ancient and 16 modern individuals and includes sequence data from four previously published ancient samples which we sequenced to higher coverage (10–18x). Such a resource will allow researchers to analyse their new samples with the same genetic pipeline and directly compare them to the reference dataset without re-processing published samples. Moreover, this dataset can be easily expanded to increase the sample distribution both across time and space.

## Abstract (summary)

**Table:** 

Measurement(s)	genome
Technology Type(s)	DNA sequencing
Factor Type(s)	modern/ancient human
Sample Characteristic - Organism	Homo sapiens

Machine-accessible metadata file describing the reported data: 10.6084/m9.figshare.14839329

## Background & Summary

The number of ancient humans with genome-wide data available has increased from less than five a decade ago to more than 3,000 thanks to advancements in extraction and sequencing methods for ancient DNA (aDNA)^1^. However, there are just a few high-quality (coverage >10x) shotgun whole-genome sequenced ancient samples^2^. While genetic pipelines have been previously published^3–6^, combining data processed with different approaches is hard and time consuming. Therefore, researchers have to download raw reads of published samples and reprocess them to create a dataset to compare their new samples against without pipeline-associated biases. This problem is less pronounced for modern DNA samples as the higher quality of DNA and sequencing coverage partially reduce the biases introduced by the usage of different bioinformatic tools.

**Fig. 1:**
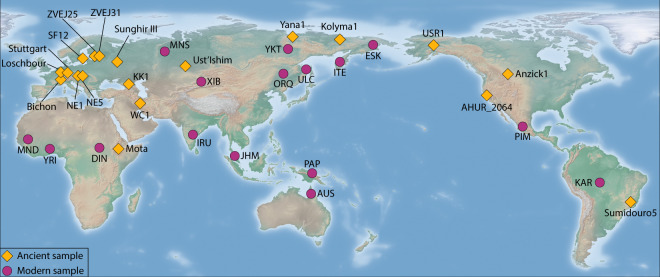
Geographic distribution of samples included in the dataset. Population acronyms are reported in Table 2.

Panels including shotgun data for modern samples distributed worldwide have been previously published, such as the Simons Genome Diversity Program^7^, 1000 Genome Project^8^ and Human Genome Diversity Project (HGDP-CEPH panel)^9^. However, the same concept has not yet been applied to ancient samples or a mix of modern and ancient samples. This study aims to start filling this gap by creating a dataset including both modern and ancient samples distributed across all continents. Therefore, we fully reprocessed 15 high-quality shotgun sequenced ancient samples downloaded from the literature, generated additional new data for previously published 4 ancient samples and merged them with 16 modern samples. The final dataset includes 35 individuals and researchers can use it to quickly compare their new samples against a set of individuals distributed across time and space (Fig. 1). Moreover, we hope that researchers will add additional data processed with the pipeline that we released to increase the sample resolution both in time and space.

## Methods

### Sample collection

Additional sequence data were generated for four ancient samples which were previously collected and described in the following original publications: ZVEJ25 and ZVEJ31 were published in Jones *et al*.^10^, KK1 in Jones *et al*.^11^ and NE5 in Gamba *et al*.^12^. Furthermore, 15 additional ancient samples and 16 modern samples have been downloaded from the literature (see Online-only Tables 1 and 2). The final dataset includes 35 samples consisting of 19 ancient and 16 modern samples.

### DNA extraction, Library preparation and next-generation sequencing

DNA was extracted and libraries were prepared for ZVEJ25, ZVEJ31, KK1 and NE5 (Table 1), following protocols described in the original publications, with the exception that DNA extracts were incubated with USER enzyme (5 µl enzyme: 16.50 µl of extract) for 3 hours at 37 °C prior to library preparation in order to repair post-mortem molecular damage. The libraries were sequenced across 31 lanes of a HiSeq. 2,500.

**Table 1:** Data statistics for newly sequenced samples.

Sample ID	Mass sampled (g)	Average autosomal coverage
Kotias (KK1)	0.101	12.03
Latvia_HG2 (ZVEJ25)	0.092	18.17
NE5 (14.6)	0.18	15.99
ZVEJ31	0.102	9.97

Average autosomal coverage was estimated on bam files after mapping quality filtering (mq20), duplicates removal, indel realignment and 2 bp softclipping.

### Bioinformatics analysis

#### Ancient samples

The following approach was used for both the newly sequenced ancient samples and downloaded raw fastq files from previously published ancient samples.

Adapters were trimmed with cutadapt v1.9.1^13^ and then raw reads were aligned to human reference sequence hg19/GRCh37 with the rCRS mitochondrial sequence using bwa aln v0.7.12^14^ with seeding disabled (-l 1000), maximum edit distance set to -n 0.01 and maximum number of gap opens set to -o 2. These parameters are recommended for aDNA as they allow for more mismatches to the reference genome^15^. Sai files were converted into sam files using bwa samse v0.7.12 and the read group line was also added. Bam files were generated using samtools view v1.9^16^. Reads from multiple libraries belonging to the same sample were merged with the module MergeSamFiles within Picard v2.9.2^17^. Aligned reads were filtered for minimum mapping quality 20 with samtools view v1.9. Indexing, sorting and duplicate removal (rmdup) were performed with samtools v1.9. Indels were realigned using The Genome Analysis Toolkit v3.7^18^ (module RealignerTargetCreator and IndelRealigner) and 2 bp were softclipped (phred quality score reduced to 2) at the start and ends of reads using a custom python script. Final bam files were split by chromosome using samtools view v1.9 and variant calling was performed with UnifiedGenotyper from The Genome Analysis Toolkit v3.7. All calls were filtered for minimum base quality 20 (−mbq 20) and reference-bias free priors were used (−inputPrior 0.0010 -inputPrior 0.4995). The same priors have been used for modern samples in the Simons Genome Diversity Panel^7^.

Raw data was not available for four previously published samples included in this dataset and so alignment data was processed instead (Loschbour, Stuttgart_LBK, Ust_Ishim and WC1). The data for Loschbour, Stuttgart_LBK and Ust_Ishim had been aligned to GRCh37 with additional decoy sequences (hs37d5) using the same non-default bwa aln parameters. We removed reads aligning to these decoys and updated the bam file headers accordingly, before proceeding with the processing pipeline outlined above. The available alignment data from WC1 was mapped using bwa aln with default parameters and had a mapping quality filter of 25 already applied. We realigned these reads using the non-default parameters and proceeded with the processing pipeline.

For those who wish to follow this pipeline with newly produced ancient DNA data, we recommend a final data authentication step. Characteristic patterns of aDNA post-mortem damage (e.g. short read lengths and cystosine deamination) can be verified using mapDamage software^19^. A number of methods exist to estimate contamination levels on the basis of these damage patterns, as well as other measures, including heterozygosity at haploid loci and the breakdown of linkage disequilibrium^20–23^

We focused on selecting a subset of the genome representing neutral genomic variation for demographic inferences^24,25^. Therefore, specific filters were applied to discard: recombination hotspots (filter_hotspot1000g), poor mapping quality regions (filter_Map20), recent duplication (recent duplications, RepeatMasker score <20), recent segmental duplication (filter_segDups), simple repeats (filter_simpleRepeat), gene exons together with 1000 bp flanking and conserved elements together 100 bp flanking (filter_selection_10000_100) and positions with systematic sequencing errors (filter_SysErrHCB and filter_SysErr.starch). All CpG sites were removed as well as C and G sites with an adjacent missing genotype. Genotypes were filtered by minimum coverage 8x and maximum coverage defined as twice the average coverage. Vcf files per chromosome belonging to the same sample were concatenated using vcf-concat from vcftools v0.1.15^2^ ^26^.

#### Modern samples

Bam files were downloaded from the Simons Genome Diversity Panel^7^ and from McColl *et al*.^27^. (Table 2). Bam files were split by chromosome and variant calling, filtering for GC sites and coverage were performed as described above for the ancient samples with the same options and thresholds.

**Table 2:** Metadata for modern samples. SGDP: Simons Genome Diversity Panel.

Sample_ID	Sample_acronym	Population_ID	Country	Latitude	Longitude	Study
SS6004477	AUS	Australian	Australia	−13	143	SGDP – Mallick *et al*., 2016
LP6005443-DNA_B09	DIN	Dinka	Sudan	8.8	27.4	SGDP – Mallick *et al*., 2016
LP6005443-DNA_B03	ESK	Eskimo_Sireniki	Russia	64.4	173.9	SGDP – Mallick *et al*., 2016
LP6005519-DNA_D05	IRU	Irula	India	13.5	80	SGDP – Mallick *et al*., 2016
LP6005443-DNA_D04	ITE	Itelman	Russia	57	157	SGDP – Mallick *et al*., 2016
LP6005441-DNA_G06	KAR	Karitiana	Brazil	−10	−63	SGDP – Mallick *et al*., 2016
LP6005441-DNA_E07	MND	Mandenka	Senegal	12	−12	SGDP – Mallick *et al*., 2016
LP6005443-DNA_G04	MNS	Mansi	Russia	63.65	62.1	SGDP – Mallick *et al*., 2016
LP6005441-DNA_F09	ORQ	Oroqen	China	50.4	126.5	SGDP – Mallick *et al*., 2016
LP6005443-DNA_D08	PAP	Papuan	PapuaNewGuinea	−4	143	SGDP – Mallick *et al*., 2016
LP6005441-DNA_F10	PIM	Pima	Mexico	29	−108	SGDP – Mallick *et al*., 2016
LP6005442-DNA_H12	ULC	Ulchi	Russia	52.43	140.42	SGDP – Mallick *et al*., 2016
LP6005442-DNA_D01	XIB	Xibo	China	43.5	81.5	SGDP – Mallick *et al*., 2016
LP6005442-DNA_F01	YKT	Yakut	Russia	63	129.5	SGDP – Mallick *et al*., 2016
LP6005442-DNA_B02	YRI	Yoruba	Nigeria	7.4	3.9	SGDP – Mallick *et al*., 2016
JHM06	JHM	Jehai	Malaysia	5.25	101.17	McColl *et al*., 2018

#### Final dataset

Per sample vcf files were compressed with bgzip and indexed with tabix from htslib v1.6^16^. The final dataset was assembled by merging filtered compressed vcf files for all modern and ancient samples with bcftools merge v1.6^16^. Only sites with called genotypes for all samples were kept using vcftools v0.1.15 (--max-missing 1). Tri-allelic sites were also discarded using bcftools view v1.6 (-m1 -M2). Final vcf statistics were generated with bcftools stats v1.6. Downstream analysis and plotting were performed in R v3.6.3^28^.

## Data Records

All newly generated sequencing raw reads have been deposited in the NCBI Sequence Read Archive Bioproject PRJNA670050^29^. Both filtered and unfiltered vcf files have been uploaded to figshare^30^.

## Technical Validation

### Summary of newly generated data

DNA was extracted for four previously published samples (ZVEJ25, ZVEJ31, KK1 and NE5) and sequence data were generated with an average coverage between 10x and 18x (Table 1). Endogenous DNA was estimated between 0.48 and 0.71 across all libraries (Table 3). Each library generated between 150 and 425 millions of reads corresponding to 15.2 and 42.9 Gb respectively (Table 3).

**Table 3:** Raw data statistics for the newly sequenced libraries.

Sample	Total Bases	Read Count	GC (%)	Q20 (%)	Q30 (%)	Reads Aligned	Endogenous DNA
KK1_1	32,085,537,489	317,678,589	49.3	96.6	94.5	226,739,842	0.71
KK1_2	31,821,488,543	315,064,243	49.7	96.9	94.8	221,241,435	0.70
KK1_3	30,903,010,501	305,970,401	47.8	96.6	94.4	218,378,529	0.71
KK1_4	28,374,056,452	280,931,252	48.5	96.6	94.5	200,616,589	0.71
KK1_5	27,051,061,997	267,832,297	47.4	96.8	94.8	187,070,443	0.70
KK1_6	26,428,490,321	261,668,221	49.7	96.7	94.5	182,602,757	0.70
NE5_1	15,230,188,243	150,793,943	48.4	96.7	94.6	113,866,866	0.76
NE5_2	22,443,822,868	222,216,068	47.8	96.7	94.6	167,444,317	0.75
NE5_3	19,414,144,957	192,219,257	47.7	96.7	94.6	145,145,785	0.76
NE5_4	35,602,627,361	352,501,261	48.9	96.8	94.7	257,297,424	0.73
NE5_5	39,509,022,440	391,178,440	49.5	96.7	94.5	285,303,006	0.73
NE5_6	38,119,633,918	377,422,118	47.7	96.8	94.7	275,284,926	0.73
ZVEJ25_1	22,502,142,793	222,793,493	48.2	96.8	94.6	173,630,441	0.78
ZVEJ25_2	26,264,479,451	260,044,351	47.5	96.8	94.6	202,756,810	0.78
ZVEJ25_3	19,884,007,259	196,871,359	48.1	96.8	94.6	153,807,348	0.78
ZVEJ25_4	30,314,118,184	300,139,784	47.0	96.9	94.8	234,102,091	0.78
ZVEJ25_5	34,172,785,511	338,344,411	48.2	96.9	94.7	264,070,011	0.78
ZVEJ25_6	32,515,172,804	321,932,404	48.2	96.9	94.7	251,187,453	0.78
ZVEJ31_1	42,951,382,412	425,261,212	52.0	96.9	94.7	215,656,479	0.51
ZVEJ31_2	41,717,115,447	413,040,747	50.7	96.9	94.8	209,910,986	0.51
ZVEJ31_3	36,806,312,233	364,418,933	53.8	96.7	94.4	185,131,989	0.51
ZVEJ31_4	34,986,764,509	346,403,609	51.3	96.9	94.6	166,115,737	0.48
ZVEJ31_5	34,797,229,121	344,527,021	53.8	96.8	94.5	164,914,158	0.48
ZVEJ31_6	39,275,860,102	388,869,902	52.0	96.8	94.6	185,999,314	0.48

### Summary of the whole dataset including ancient and modern samples

**Fig. 2:**
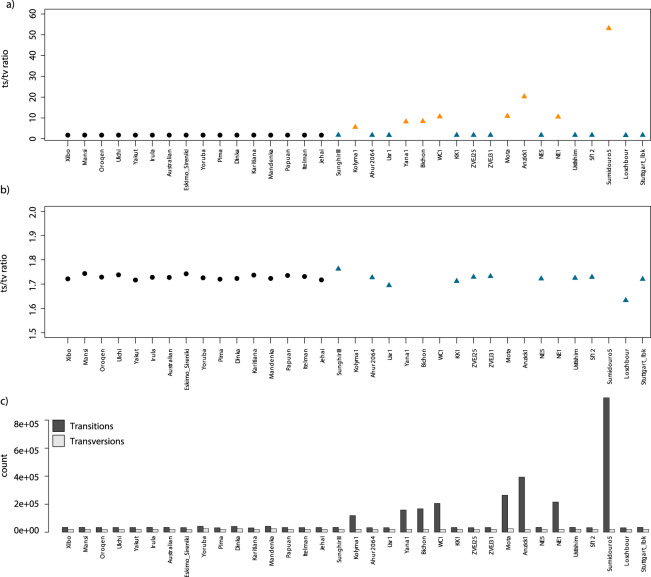
(**a**) Transitions/Transversions ratio (ts/tv) per sample. Ancient and modern samples are represented by triangles and circles respectively. UDG and non-UDG treated samples are in blue and orange respectively. (**b**) same as in a) but with a different y axis to focus on the ts/tv ratio among modern and UDG-treated ancient samples. (**c**) Number of transitions (ts) and transversions (tv) per sample.

The final dataset includes 35 samples with 509,351,727 sites in neutral regions before filtering (see Methods section for a detailed description of which regions were considered for variant calling). Sites not called across all samples (0% missing data allowed) were then discarded and 72,045,170 were retained. Multi-allelic sites (3815) were also removed bringing the final number of filtered sites to 72,041,355 (Online-only Table 2). Minimum and maximum coverage per sample within the final dataset is 11.3x and 55x respectively (within filtered intervals) with an average coverage across all samples of 29.7x (Online-only Table 2). We calculated the number of transitions (ts), transversions (tv) and the ts/tv ratio per sample (Online-only Table 2). As expected, all eight ancient samples that were not subjected to UDG-treatment showed a higher ts/tv ratio than their UDG-treated counterparts (see Fig. 2), consistent with higher levels of DNA damage in these samples. The Brazilian sample Sumidouro 5 shows the highest excess of transition, possibly due to poor DNA preservation caused by environmental conditions. All other samples (both modern and UDG-treated ancient) showed similar ts/tv ratio with an average of 1.72, maximum and minimum of 1.76 and 1.63 respectively (see Online-only Table 2, Fig. 2).

## Data Availability

All newly generated sequencing raw reads (see Table 3) have been deposited in the NCBI Sequence Read Archive (SRR12854172, SRR12854173, SRR12854174, SRR12854175). Six compressed fastq files per sample were uploaded. The fastq files have the same names as the libraries described in Table 3. The genetic pipeline used to process the data is available at https://github.com/EvolEcolGroup/data_paper_genetic_pipeline. The filtered compressed vcf file used for the analyses has been uploaded to figshare^30^ with the title “A curated dataset of modern and ancient high-coverage shotgun human genomes”.

## Online-only Tables

**Online-only Table 1:** Metadata for ancient samples. Samples in bold have been resequenced in this study.

Sample	Study	County	Site	Latitude	Longitude	Mean date BP	Date (2-sigma)	UDG-treated
AHUR_2064	Moreno-Mayar JV *et al*., 2018	USA	Spirit Cave, Nevada	37.41	−122.08	10970	10770–11170 calBP	yes
Anzick-1	Rasmussen M *et al*., 2014	USA	Near Wilsall, Montana	45.97	−110.66	12632	12707–12556 calBP	no
Bichon	Jones *et al*. 2015	Switzerland	Bichon	47.1	6.87	13665	13560- 13770 cal BP	no
**KK1**	Jones *et al*. 2015	Georgia	Kotias Klde	42.25	43.27	9712	9529–9895 cal BP	yes
Kolyma1	Sikora M *et al*., 2019	Russia	Duvanni Yar	68.6	159.1	9786	9668–9904 calBP	no
Loschbour	Lazaridis *et al*. 2014	Luxembourg	Echternach	49.81	6.4	8055	6220–5990 calBCE	yes
Mota	Gallego-Llorente M *et al*.,2015	Africa	Mota Cave, Gamo highlands of southwest Ethiopia	6.80	38.17	4471	4524–4418 Cal BP	no
NE1	Gamba *et al*. 2014	Hungary	Polgar Ferenci hat	47.88	21.19	7140	5310-5070 calBC	yes
**NE5**	Gamba *et al*. 2014	Hungary	Kompolt-Kigyoser	47.17	20.83	7050	5210-4990 calBC	yes
SF12	Guenther *et al*. 2018	Sweden	Stora Förvar, Sweden	57.28	18	7700	7500-4000 cal BC	yes
Sumidouro5	Sikora *et al*. 2017	Brazil	Caverna do Sumidouro, Lagoa Santa, Brazil	−19.54	−43.94	10391	10258–10524 (97.0%) calBP	no
sunghirIII	Moreno-Mayar JV *et al*., 2018	Russia	Sunghir	56.176	40.503	34093	35154-33031 calBP	yes
USR1	Moreno-Mayar JV *et al*., 2018	USA	Upward Sun River site (USR)	64.98	−150.54	11435	11600-11270 cal BP	yes
Ust_Ishim	Fu *et al*. 2014	Russia	Ust’-Ishim, Omsk Oblast	57.43	71.1	45000	45000 calBP (46880–43210 calBP at 95.4% probability)	yes
WC1	Broushaki *et al*. 2016	Iran	Wezmeh Cave	34.05	46.59	9219	7455-7082 BCE	no
Yana1	Sikora M *et al*., 2019	Russia	Yana RHS	70.43	135.25	31684	31321–32047 calBP	no
**ZVEJ25**	Jones *et al*., 2017	Latvia	Zvejnieki	57.78	25.24	7689	7791-7586 calBP	yes
**ZVEJ31**	Jones *et al*., 2017	Latvia	Zvejnieki	57.78	25.24	5965	6179-5750 calBP	yes
Stuttgart_LBK	Lazaridis *et al*. 2014	Germany	Viesenhäuser Hof, Stuttgart-Mühlhausen	48.78	9.18	7143	5308-5077 cal BC	yes

**Online-only Table 2:** variant calling summary per sample. DP: depth of coverage in filtered intervals for variant calling.

Sample	Ref_Hom_sites	Alt_Hom_sites	Het_sites	Transitions (ts)	Transversions (tv)	Average_DP	ts/tv ratio
Xibo	71987288	22400	31667	34202	19865	36.6	1.72
Mansi	71987458	21384	32513	34253	19644	45.6	1.74
Oroqen	71988238	23119	29998	33652	19465	39	1.73
Ulchi	71987979	23038	30338	33883	19493	42	1.74
Yakut	71987674	22678	31003	33922	19759	38.1	1.72
Irula	71986543	21446	33366	34720	20092	52.8	1.73
Australian	71987399	24959	28997	34174	19782	43.5	1.73
Eskimo-Sireniki	71989128	23323	28904	33184	19043	43.6	1.74
Yoruba	71973954	22014	45387	42675	24726	34.3	1.73
Pima	71990719	24773	25863	32022	18614	36.3	1.72
Dinka	71975528	22325	43502	41656	24171	36	1.72
Karitiana	71992172	25354	23829	31214	17969	44.2	1.74
Mandenka	71974397	22203	44755	42372	24586	33.2	1.72
Papuan	71988501	25961	26893	33533	19321	41.6	1.74
Jehai	71987596	23346	30413	33976	19783	36	1.72
Itelman	71988890	24013	28452	33256	19209	47.1	1.73
SunghirIII	71987765	23614	29976	34194	19396	13.5	1.76
Kolyma1	71901819	23824	115712	118330	21206	16.3	5.58
AHUR_2064	71990711	24368	26276	32074	18570	20	1.73
USR1	71989335	24257	27763	32717	19303	19.5	1.69
Yana1	71863578	22613	155164	158463	19314	28.8	8.20
Bichon	71854465	23232	163658	166961	19929	11.3	8.38
WC1	71816753	21018	203584	205313	19289	12	10.64
KK1	71988156	22438	30761	33585	19614	15.7	1.71
ZVEJ25	71989802	22913	28640	32665	18888	23.2	1.73
ZVEJ31	71988236	22147	30972	33678	19441	13.5	1.73
Mota	71753225	22609	265521	263875	24255	13.6	10.88
Anzick-1	71628867	22549	389939	393092	19396	15.4	20.27
NE5	71987353	21382	32620	34165	19837	20.8	1.72
NE1	71805092	20915	215348	215777	20486	23.9	10.53
UstIshim	71986388	21569	33398	34796	20171	35.2	1.73
Sf12	71989990	22548	28817	32544	18821	55	1.73
Sumidouro5	71064691	20902	955762	958624	18040	16.2	53.14
Loschbour	71990147	24525	26683	31762	19446	19.3	1.63
Stuttgart_LBK	71987124	21496	32735	34298	19933	17.1	1.72
